# Supplementary material for: No cognitive or psychological impact from returning research Alzheimer disease biomarkers: A delayed-start, noninferiority, randomized clinical trial
Source: medRxiv. 2026 Jun 10:2026.05.22.26353881. Preprint. [Version 2] doi: 10.64898/2026.05.22.26353881 (PMC13278217; doi:10.64898/2026.05.22.26353881)

medRxiv preprint doi: https://doi.org/10.64898/2026.05.22.26353881; this version posted June 10, 2026. The copyright holder has placed this preprint (which was not certified by peer review) in the Public Domain. It is no longer restricted by copyright. Anyone can legally share, reuse, remix, or adapt this material for any purpose without crediting the original authors.

Supplemental Figure 1: Integrated WeSHARE study flow diagram

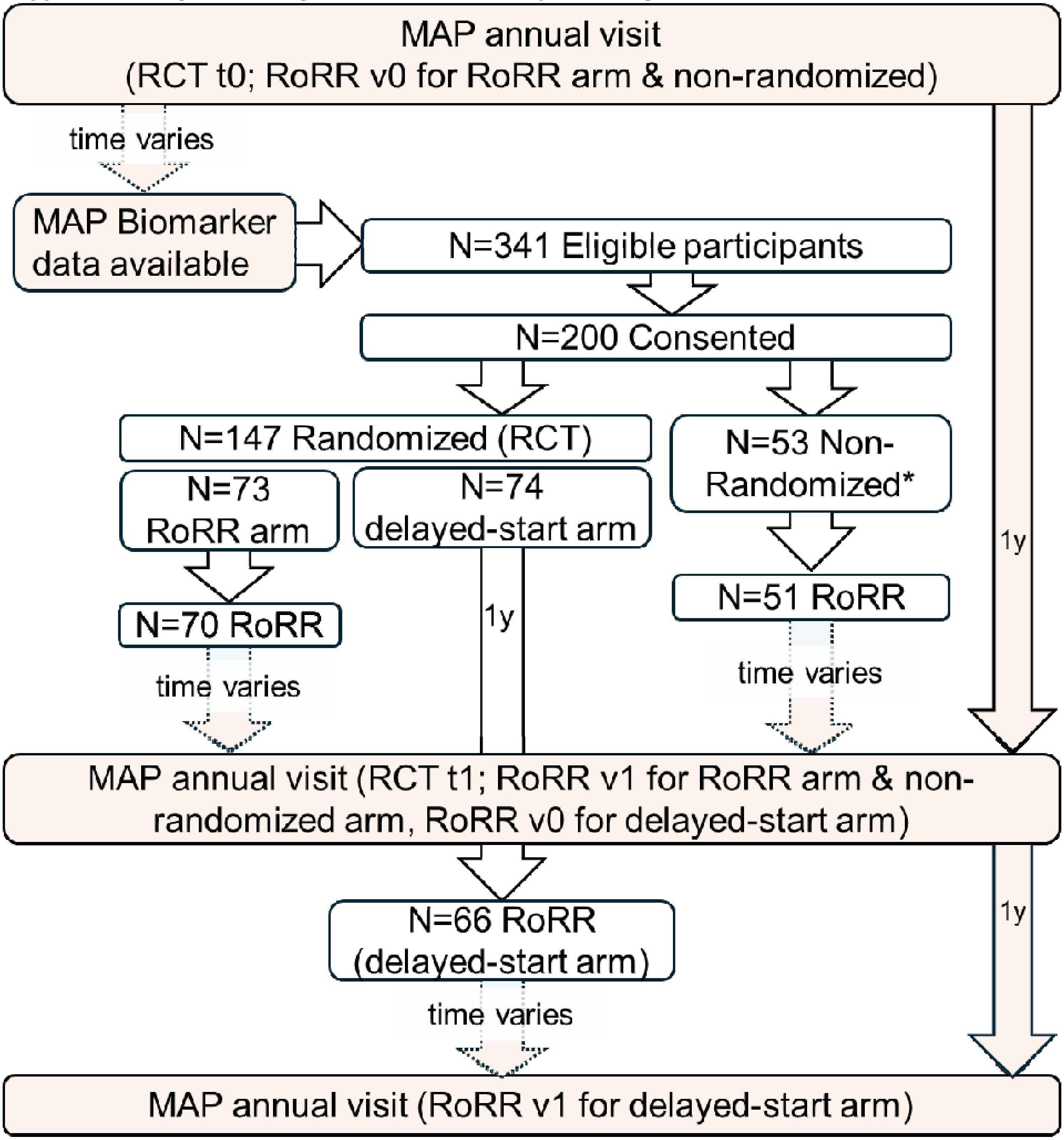

MAP: Memory & Aging project; parent longitudinal study
RoRR: Return of research results
*Part of pilot study or unable to be randomized; same processes as RoRR arm

medRxiv preprint doi: https://doi.org/10.64898/2026.05.22.26353881; this version posted June 10, 2026. The copyright holder has placed this preprint (which was not certified by peer review) in the Public Domain. It is no longer restricted by copyright. Anyone can legally share, reuse, remix, or adapt this material for any purpose without crediting the original authors.

Supplemental Figure 2: Observed changes in outcome based on proximity from RoRR to follow-up outcome measurement GDS (A), CDR-SB (B), cognitive composite (C), subjective cognitive impairment (D).

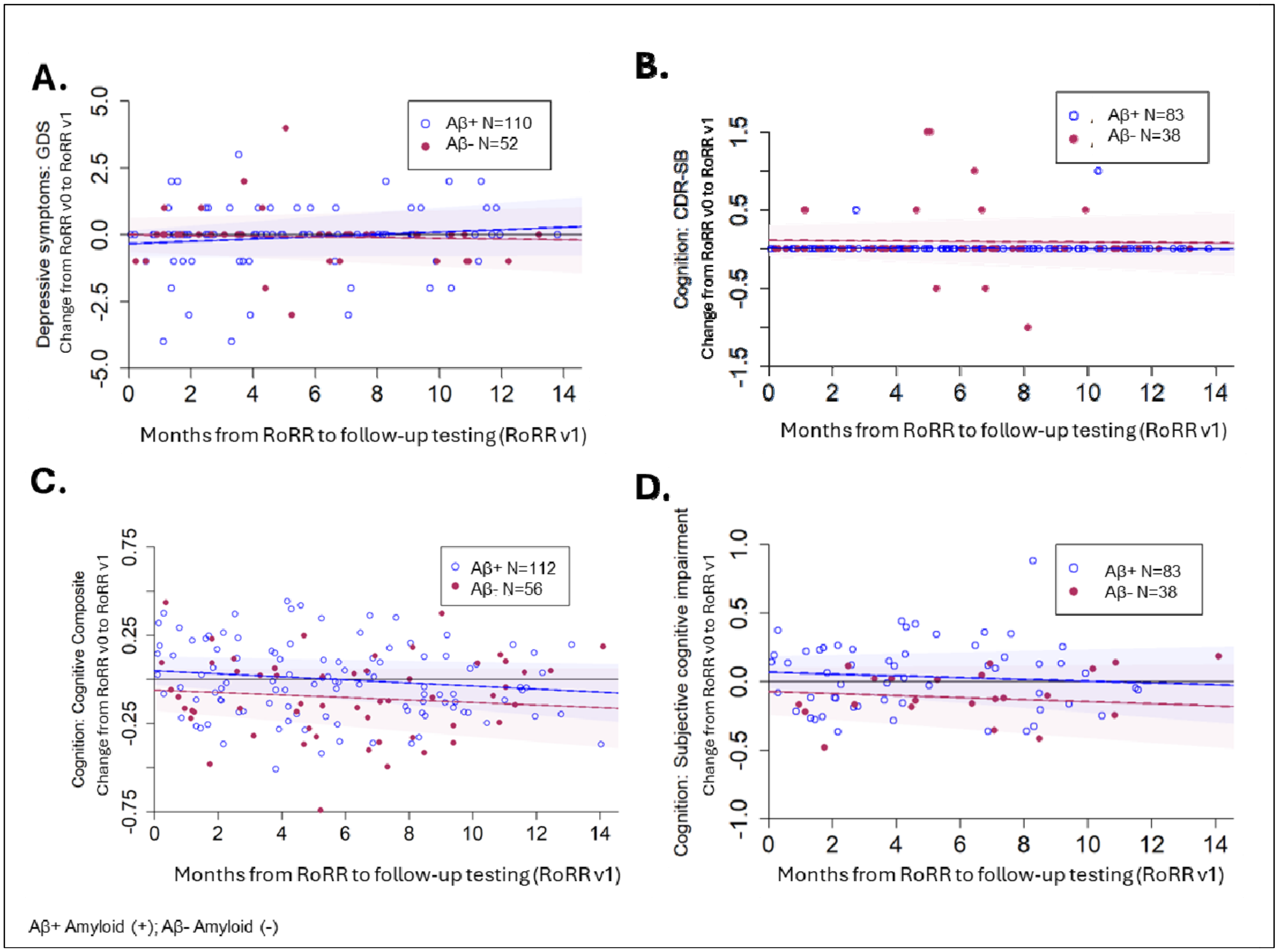

Supplement: Supplementary file 1 [file NIHPP2026.05.22.26353881v2-supplement-1.pdf]
